# JMJD3 regulate H3K27me3 modification via interacting directly with TET1 to affect spermatogonia self-renewal and proliferation

**DOI:** 10.1186/s12864-024-10120-9

**Published:** 2024-02-29

**Authors:** Jin Wang, Lingling Liu, Zebin Li, Haoyu Wang, Yuanyuan Ren, Kaisheng Wang, Yang Liu, Xinjie Tao, Liming Zheng

**Affiliations:** School of Basic Medical Sciences, Anhui Medical University, Hefei, China

**Keywords:** Spermatogonia, TET1, JMJD3, H3K27me3, Pramel3

## Abstract

**Background:**

In epigenetic modification, histone modification and DNA methylation coordinate the regulation of spermatogonium. Not only can methylcytosine dioxygenase 1 (TET1) function as a DNA demethylase, converting 5-methylcytosine to 5-hydroxymethylcytosine, it can also form complexes with other proteins to regulate gene expression. H3K27me3, one of the common histone modifications, is involved in the regulation of stem cell maintenance and tumorigenesis by inhibiting gene transcription.

**Methods:**

we examined JMJD3 at both mRNA and protein levels and performed Chip-seq sequencing of H3K27me3 in TET1 overexpressing cells to search for target genes and signaling pathways of its action.

**Results:**

This study has found that JMJD3 plays a leading role in spermatogonia self-renewal and proliferation: at one extreme, the expression of the self-renewal gene GFRA1 and the proliferation-promoting gene PCNA was upregulated following the overexpression of JMJD3 in spermatogonia; at the other end of the spectrum, the expression of differentiation-promoting gene DAZL was down-regulated. Furthermore, the fact that TET1 and JMJD3 can form a protein complex to interact with H3K27me3 has also been fully proven. Then, through analyzing the sequencing results of CHIP-Seq, we found that TET1 targeted Pramel3 when it interacted with H3K27me3. Besides, TET1 overexpression not only reduced H3K27me3 deposition at Pramel3, but promoted its transcriptional activation as well, and the up-regulation of Pramel3 expression was verified in JMJD3-overexpressing spermatogonia.

**Conclusion:**

In summary, our study identified a novel link between TET1 and H3K27me3 and established a Tet1-JMJD3-H3K27me3-Pramel3 axis to regulate spermatogonia self-renewal and proliferation. Judging from the evidence offered above, we can safely conclude that this study provides new ideas for further research regarding the mechanism of spermatogenesis and spermatogenesis disorders on an apparent spectrum.

**Supplementary Information:**

The online version contains supplementary material available at 10.1186/s12864-024-10120-9.

## Background

Epigenetic inheritance refers to the modification of chromosomal genes without altering the DNA sequence, resulting in certain changes in the human phenotype [1]. Epigenetic regulation plays a key role in cell proliferation, differentiation, embryonic development and stem cell maintenance [2]. Epigenetic inheritance includes DNA methylation and histone modification, in which the basic constituent units of chromatin are mainly nucleosomes composed of DNA and proteins encircling them, whose function is to participate in the transcriptional regulation of genes, with the more studied proteins including histones [3]. Histone modification is mainly used to regulate the expression of important genes by modifying terminal lysine residues of different proteins to convert them into protamine. Four subtypes exist in nucleosomes, H2A, H2B, H3 and H4, with H3 being the most widely modified [4, 5]. H3K4 is associated with the activation of gene transcription, whereas H3K9 and H3K27 are enriched at the promoters of silenced genes and are closely linked to gene transcription suppression [1]. H3K27me3 is suppressive of histone modification, mainly catalyzed by Polycomb Repressive Complex 2 (PRC2) [6]. EZH2(enhancer of zeste homolog 2) causes the selective repression of gene transcription by promoting H3K27me3 [7]. EZH1(enhancer of zeste homolog 1) partially antagonizes the role of EZH2 in H3K27me3 [8]. JMJD3 is a demethylase, also known as lysine-specific demethylase 6B (KDM6B), which belongs to the JMJC family of proteins, reducing the transcriptional repression of H3K27me3 by demethylation and contributing to the activation of transcription [9]. JMJD3 regulates the proliferation of undifferentiated spermatogonia and plays a role in stem cells and development [10, 11].

Many reports suggest that regulation of the balance between DNA methylation and histone modifications plays a crucial role in spermatogonia homeostasis [12–14]. DNA methylation is the recognition of CpG by DNA methyltransferases and the addition of methyl groups to its position to turn it into 5-methylcytosine; methylation and demethylation work together to maintain methylation homeostasis [15]. The TET1 protein is a 5-methylcytosine hydroxylase belonging to the TET protein family, which participates in DNA demethylation through its N-terminal CXXC structural domain binding to promoter regions [16]. The TET family participates in crosstalk between H3K27me3 and DNA methylation regulation in mouse embryonic cells and spermatogenesis [17]. It has been reported that the regulatory function of TET1 in early ESC cell development is mainly mediated through its non-catalytic role in the establishment of bivalence of the developmental genes H3K4me3 and H3K27me3 and the prevention of their premature activation [18]. Studies on the non-catalytic role of TET1 have revealed that it interacts with several protein complexes, including EZH2 in H3K27me3 and the SIN3A protein [19, 20]. TET1 interacts with polycomb repressive complex 2 (PRC2) to form a histone modification complex that modifies the chromatin repressor (H3K27me3) in mouse embryonic stem cell to regulate transcription [21].

Spermatogenesis is a very complex process consisting of three stages: mitosis, where the self-renewal and differentiation of spermatogonia occurs; meiosis, where spermatocytes undergo two meiotic divisions to form spermatozoa; and sperm metamorphosis, where spermatozoa undergo differentiation to form mature spermatozoa [22]. Spermatogonia maintains a sufficient number of cells through continuous self-renewal, proliferation and differentiation; this is an important step to ensure normal spermatogenesis [23]. The mechanisms that regulate the critical stages of spermatogenesis are still largely unknown. Spermatogenesis is a coordinated process of various mechanisms that requires the tight regulation of gene expression by transcription factors and epigenetic modifiers [24]. Chromatin remodeling is required during the self-renewal and differentiation of spermatogonia, which resets the balanced chromatin structure, including DNA methylation and histone modifications. They maintain transcriptional regulatory balance by initiating a series of genes [25]. PRC2 mediates cellular self-renewal, and differentiation is significantly expressed at the developmental promoter of H3K27me3 in male germ cells [26]. H3K27me3 and PRC2 have been extensively studied in mouse embryonic stem cell pluripotency and are localized to the promoters of genes that repress development; it has been reported that all PRC2 components are expressed to some extent during germ cell development, with PRC2 core component activity being critical for spermatocyte and oocyte development [27]. It has been suggested that TET1 has a potential role in the PRC2-mediated inhibition of developmental regulators [13]. TET1 regulates methylation homeostasis by forming complexes with various methylating and demethylating enzymes of H3K27me3 to maintain the balance between spermatogonia self-renewal and differentiation [28], but the specific mechanism of action of TET1 and H3K27me3 regulation in spermatogonia has not been investigated to a greater depth. The Pramel3 protein plays an important role in spermatogenesis and is detected in spermatogonia, early spermatocytes (anterior fine line, fine line and syncytial line) and round spermatocytes, which are evenly distributed in the cytoplasm and nucleus [29]. Pramel3 belongs to the family of PRAME(preferentially expressed antigen of melanoma) genes, which act as a cancer/testis antigen that is mainly expressed in the normal testis and is also implicated in immunity and reproduction; deletion of the PRAME family of genes leads to smaller testes and a reduction in the number of spermatozoa produced [30]. PRAME acts through the retinoic acid receptor (RAR) signaling pathway in melanoma and other cancer cells and RA signaling is necessary for male fertility [30–32].

Determining the specific regulatory mechanisms of TET1 and H3K27me3 in spermatogonia self-renewal, proliferation and differentiation is conducive to further resolution of the causes of spermatogenic disorders in males and provides a greater scientific basis for solving the problem of male infertility. In this study, we hypothesized that the overexpression of TET1 in spermatogonia and its coordinated role with JMJD3 regulates changes in H3K27me3 content, which ultimately work together to regulate spermatogonia self-renewal and proliferation. To test this hypothesis, we examined JMJD3 at both mRNA and protein levels and performed Chip-seq sequencing of H3K27me3 in TET1 overexpressing cells to search for target genes and signaling pathways of its action. Collectively, our evidence suggests that TET1 affects the methylation content of H3K27me3 and reduces the repression of Pramel3 expression to co-regulate the dynamic balance of spermatogenesis.

## Materials and methods

### Cell culture

The spermatogonia GC-1 and 293T cells were purchased from the American Typical Collection of Biological Resources (ATCC, Rockville, MD, USA), which were cultured in DMEM basal medium (319-005-CL, WISENTINC) supplemented with 10% fetal bovine serum (EVERY GREEN) in a humidified environment at 37℃ with 5% carbon dioxide [23, 33, 34].

### Lentiviral concentration/transduction and positive cell clone screen

#### Lentiviral concentration

When 293T cells were grown at 75% density, fresh DMEM consisting of all supplements was replaced, and after 30 min, 8ug of the target plasmids PCDH-EZH2, PCDH-JMJD3 and the control plasmid PCDH (Additional file 1: Fig. 1A) were added to 100ul of DMEM with PAX and VSVG in the ratio of 4:3:2, respectively, and mixed, incubate at room temperature for 20 min and then add to 293T cell culture dish. Observe the green fluorescence under the microscope after 24 h of incubation, collect the virus supernatant after 48 h of incubation, for every 30 ml of filtered initial virus solution, add 5XPEG-8000 NaCl master batch 7.5 ml. 4 °C, 4000 g, centrifugation for 20 min, add the appropriate amount of lentivirus lysate to dissolve the lentivirus precipitate, the virus suspension after concentration is divided into 50 µl per portion, stored in the finished product tubes, the stored at -80 °C after quick-freezing with crushed dry ice.

### Lentiviral transduction and positive cell clone screen

The collected PCDH-EZH2, PCDH-JMJD3, PCDH virus concentrates were added into normal cultured GC-1 cells respectively, and some of the cells expressed GFP green fluorescence after transduction for 24 h. Subsequently, cell clones stably transducted with PCDH-EZH2, PCDH-JMJD3, PCDH, lentiviral vectors were screened with puromycin (1000 ng/ml), and the single-cell clone was isolated after a week of dilution and culture until positive cell clone were screened (Additional file 1: Fig. 1B).

### Quantitative RT-PCR (qRT-PCR)

Total RNA was extracted from cells transfected for 24 h with an RNA extraction kit (AG21023). The cDNA was prepared with a transcription kit (BL699A, biosharp). The cDNA was then amplified by PCR using SYBR Green qPCR Mix as well as specific primers. Experiments were performed with a fluorescent quantitative PCR instrument (LC480II, Roche) using the primer sequences shown in Table 1.

**Table 1:** Primer of PCR

Gene	Forward	Reverse
GAPDH	TGGCCTTCCGTGTTCCTAC	GAGTTGCTGTTGAAGTCGCA
TET1	GAGCCTGTTCCTCGATGTGG	CAAACCCACCTGAGGCTGTT
PLZF	CACCGCAACAGCCAGCACTAT	CAGCGTACAGCAGGTCATCCAG
GFRA1	GACCGTCTGGACTGTGTGAAAG	TTAGTGTGCGGTACTTGGTGC
PCNA	AGTGGAGAACTTGGAAATGGAA	GAGACAGTGGAGTGGCTTTTGT
CylinA	TGGCTGTGAACTACATTGA	ACAAACTCTGCTACTTCTGG
VASA	GATAATCATTTAGCACAGCCTC	GTCAACAGATGCAAACACAG
DAZL	ATGTCTGCCACAACTTCTGAG	CTGATTTCGGTTTCATCCATCCT
C-KIT	CGCCTGCCGAAATGTATG	TCAGCGTCCCAGCAAGTC
EZH2	TTGCTGCTGCTCTTACTG	CACTCCACTCCACATTCTC
JMJD3	TTCCTGTTTACCGCTTCGT	CGTTCCACTCATATCGCTCC
Mbnl1	ACTTGCTCACGACCAGAC	ATTCCGCCCATTTATCT
Fthl17c	GGATGCTATCAACACCCAC	AGCCTCCACGCTTATTCT
Mtcp1	GTGGTGGAAGAGGAGACA	AGAGGTAGTTGGGAGGTAAG
Pramel3	TGGGAAGATTGTGAGCC	GGACAGGGATGGGATAG
Baat	CACTGAAAGATGAGAAGGGAA	AGGGAGGACTGACGACTATGT
H3c6	CGCAAGCTGCCGTTCCA	GTCCTTGGGCATGATGGTGA
Fcer1a	GCCACCGTTCAAGACAG	TTGCGGACATTCCAGTT
Prl2c1	TGGATACTGCTCCTACTACTG	CTGGCTTGTTCCTTGTTT
2610002M06Rik	AGCACTACTACCCTTACGA	ATCACGAAGACGAGCAA
Nedd1	GTTACATTGCTTCTGGGTC	CGGATAGGCTGCTTACTC
Gm13871	TATGAACTCGGAGACCCA	GTGCCCATCAAACCAAA
Tent5d	TAACAACGGGAAGAACG	CTTGGCATTTGGGACAC

### Western blot

Proteins were extracted from stably transfected cells for 48 h and then protein concentrations were detected using the BCA Protein Concentration Assay Kit (P0010S, Beyotime). After heat denaturation in 5% SDS-PAGE loading buffer, protein samples were separated by SDS-PAGE and transferred to PVDF membranes. Proteins were analyzed with anti-β-actin (1:10000, 66009-1-Ig, Proteintech), anti-EZH2 (1:1000, 21800-1-AP, Proteintech), anti-JMJD3 (1:1000, A01309, BOSTER), anti-H3 (1;1000, A12477-2, BOSTER), anti-H3K27me3 (1:1000, #9733, CST), anti-PCNA (1:1000, BS6438, Bioworld), anti-DAZL (1:1000, A02069-2, BOSTER), anti-GFRA1(1:1000, PB0199, BOSTER) anti-AKT (1:1000, T55561, Abmart), anti-P-AKT (1;1000, T40067, Abmart). Secondary antibodies were horseradish peroxidase-coupled anti-rabbit IgG (1:5000, SA00001-2, Proteintech) and anti-mouse IgG (1;10,000, BA1050, BOSTER). Detection was performed using an ultrasensitive ECL chemiluminescent substrate (BL523A, biosharp) and results were analyzed using a Tanon-5200 automated gel imaging system.

### Fluorescence activated cell sorter (FACS) assay

The sample cells were collected and treated using the instructions of the Cell Cycle Assay Kit (BB-4104-20T); the number of cells was 5-10 × 10^6^, which were washed twice with cold PBS and resuspended with 500 µl of PBS. Next, 3.5 ml of cold ethanol was added dropwise and fixed at -20 °C; for 1 h, before centrifuging and discarding the supernatant to collect the cells. Cells were washed twice with cold PBS, resuspended with 500 µl of cold PBS, treated with the addition of 20 µl of RnaseA solution in a water bath at 37 °C for 30 min and filtered with a 400-mesh cell sieve. The cells were collected by centrifugation and the supernatant was discarded and the cells were resuspended with 400 µl of PI solution before being incubated at 4 °C for 1 h under light protection. The data were detected by flow cytometry (bd celesta) and analyzed by FlowJo software.

### Bisulfite sequencing assay

Genomic DNA was extracted from the modified cell samples and subjected to sodium bisulfite treatment using the EZ DNA Methylation-Direct™ Kit (Zymo Research) in accordance with the instruction manual. Each DNA sample was transferred to 20 µl digestion mixture. After incubation for 3 h at 50 °C, the digested samples were treated with the addition of 130 µl CT Conversion Reagent for bisulfite conversion and incubated at 98 °C for 8 min and then at 64 °C for 3.5 h. Modified DNA was then desalted, purified and finally eluted with 15 µl of elution buffer. Subsequently, Bisulfite Sequencing PCR (BS-PCR) was immediately carried out using 2 µl of modified DNA per PCR run. Primers for EZH2 and the JMJD3 promoter are listed in Table 2. We analyzed within the front of 2000 bp of the EZH2 and JMJD3 promoter regions. Gene ID of EZH2 is 14,056, the first exon is located at 2001–2192 bp, selected the front of promoter regions at 1610–1944 bp, the EZH2 gene fragment contains 19 CpG sites. Positions in the sequence are as follows: 3, 6, 48, 54, 64, 81, 96, 105, 112, 117, 139, 147, 164, 186, 202, 205, 217, 227, 238. Gene ID of JMJD3 is 216,850, the first exon is located at 2169-2346 bp, selected the front of promoter regions at 49-370 bp, the JMJD3 gene fragment contains 18 CpG sites. The positions in the sequence are as follows: 22, 24, 52, 62, 65, 67, 76, 104, 111, 149, 157, 159, 178, 193, 204, 241, 248, 253. The BS-PCRswere performed using the Hot Start DNA polymerase Zymo Taq™ premix (Zymo Research) and the PCR system was as follows: 4 min at 95 °C, followed by 40 cycles of denaturation for 30 s at 95 °C, annealing for 30 s at 46 °C, extension for 20 s at 72 °C and a final extension at 72 °C for 7 min. The PCR products were gel-purified and subcloned into pMD18-T vectors (TaKaRa) and the clones confirmed by PCR were selected for DNA sequencing (BGI). Bisulfite sequencing data and the C–T conversion rate was analyzed by BIQ Analyzer software. Methylation data from bisulfite sequencing were evaluated by computing the percentage of methylated CPGs [14].

**Table 2:** Primer of bisulfite sequencing assay

Gene	Forward	Reverse
EZH2	GGGGTGAGTTATTTGTTTTTAAAAG	CGCCTAAACCAAATTTAAAATAATT
JMJD3	TTCGTTTTTTTTTGTTTTTTTAGAG	TCCCAACCCACATTTCTAACTAT

### Co-immunoprecipitation assay

In the co-immunoprecipitation (Co-IP) assay, mtet1 transfected into 293T cells. Forty-eight hours after transfection, cells were collected, washed three times with frozen PBS and then lysed with PAPI lysis buffer (Beyotime) containing protease inhibitor (Beyotime). Then 200 µl was removed as Input and stored at -20 °C for use. Meanwhile, the remaining protein was divided into two equal parts. Here, 400 µl of anti-TET1 antibody (40 µg/ml, GTX124207, GeneTex) and an equal amount of anti-mouse IgG (BOSTER) were added to the Protein A agarose beads (MCE) and incubated with low-speed rotation at 4 °C for 2 h. The collected sample cells were then added to the mixture and incubated with low-velocity spinning at 4 °C for 2 h. Then, the protein mixture was washed three times with 400 µl of washing buffer and separated from the magnetic beads; the supernatant was discarded, before 40 µl 1xSDS-PAGE Loading Buffer was added to the magnetic beads and mixed well, and then heated at 95 °C for 5 min. The magnetic beads were separated, the supernatant was discarded and a western blot assay was performed.

### Chip-seq data

Cells transfected with TET1 overexpression plasmid and control plasmid for 48 h were collected and subjected to Chip-seq, which consists of cell cross-linking, cell lysis, protein immunoprecipitation, extraction of co-precipitated complex DNA and construction of DNA secondary sequencing libraries. The chip-seq data discussed in this article have been deposited at NCBI and are available under GEO serial registration number GSE233150, with the link (https://www.ncbi.nlm.nih.gov/geo/query/acc.cgi?acc=GSE233150).

### Hematoxylin-eosin staining

Tissue sections were dewaxed and hydrated (with xylene for 10 min twice, anhydrous ethanol and 95%, 85% and 70% ethanol each for 5 min), washed three times in PBS for 5 min, stained with hematoxylin for 0.5–1 min, rinsed for 2 h, stained with eosin for 5 min and finally dehydrated in a gradient and blocked with a neutral gum. Pictures were taken with an inverted fluorescence microscope (DM6B).

### Immunohistochemistry

Tissue sections were first dewaxed and hydrated, then permeabilized with 0.5% Triton X-100 for 30 min at room temperature, immersed in 0.1 mol/L sodium citrate repair solution, microwaved for 10 min to a slight boil, stopped heating and then cooled naturally for 30 min to room temperature. Endogenous peroxidase blocker from the Immunohistochemistry Kit was added and solutions were incubated for 10–15 min at room temperature. JMJD3 (1:150, A01309, BOSTER) Antibody, H3K27me3 (1:150, #9733, CST) Antibody and PCNA (1:150, BS6438, Bioworld) Antibody were incubated at 4 °C overnight. The appropriate amount of reaction enhancer was added dropwise and incubated at room temperature for 30 min; a horseradish peroxidase-labeled sheep anti-rabbit/mouse IgG marker was added, incubated at 37 °C for 45 min and then an appropriate amount of prepared DAB color developer was added. Solutions were left at room temperature for 15 s and then rinsed and re-stained was in hematoxylin for 10–30 s. The sections were rinsed in tap water to return to the blue and finally dehydrated and sealed. Pictures were acquired with an inverted fluorescence microscope (DM6B).

### Animal model preparation and cellular testicular transplantation

Preparation of the busulfan model mice: Male Kunming mice of 25 g or more were selected for breeding. We weighed 60 mg of Bactrim powder (Sigma) and dissolved it in 10 mL of DMSO to prepare a Bactrim working solution at a concentration of 6 mg/mL. The mice were weighed and the working solution was injected into the peritoneal cavity of the mice at a ratio of 5 µl/g body weight; the preparation of the model mice was completed after 3 weeks.

For the anesthesia protocol in mice surgery, 30–35 g mice were selected, the mice were fixed the tail vein was sterilized and the anesthetics could be pushed in if there was blood in the retracted blood; the anesthetics used was Shutai 50 at a ratio of 0.5 ml/kg body weight. The anesthesia lasted for one hour, which was sufficient for the duration of the surgery for the mice.

Cell transplantation: Transplantation was performed in busulfan model mice by opening the peritoneal cavity of mice under general anesthesia and aseptic conditions, pulling out one side of the testis and transplanting the JMJD3 positive cell clone mixed with Trypan Blue at a ratio of 2 × 10^7^/ml and a suspension of control cells into the seminiferous tubules; the transplantation process was performed using a multipoint scatter injection of testicular tissue until approximately 90% of the seminiferous tubules were blue [35].

### Statistical analyses

Data were analyzed using GraphPad software and all data were expressed as means ± SEM. The t-test was used for appropriate comparisons between the two groups. One-way ANOVA was used to determine the significance between multiple groups. Statistical significance was defined as *p* < 0.0001 (****), *p* < 0.001 (***), *p* < 0.01 (**), *p* < 0 0.05 (*). All data were collected from three independent experiments.

## Results

### Overexpression of JMJD3 in spermatogonium promotes self-renewal

**Fig. 1:**
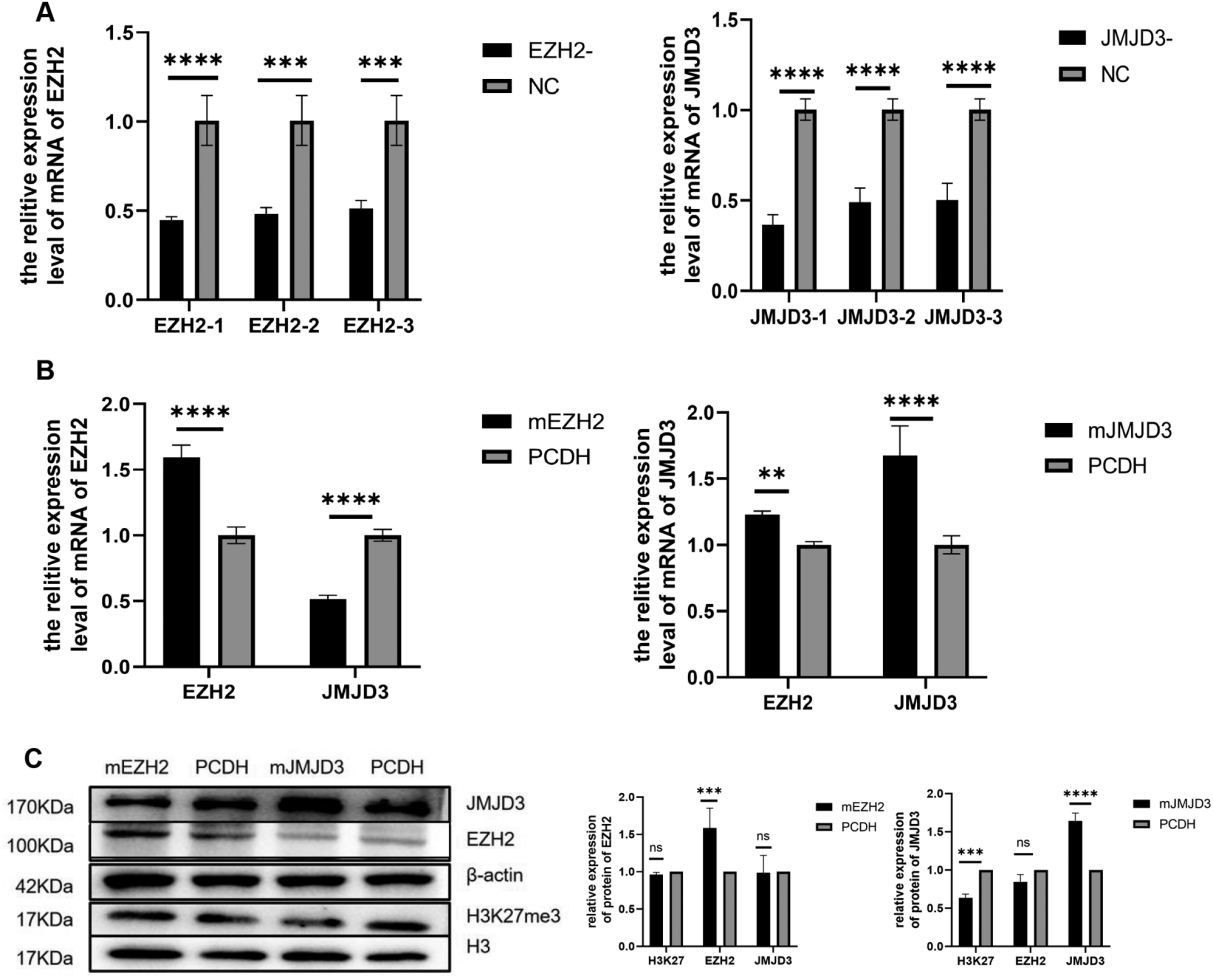
Expression levels of EZH2, JMJD3, H3K27me3 in spermatogonia. (**A**) qRT-PCR was used to detect the expression of EZH2 siRNA and JMJD3 siRNA in spermatogonia after interference. (**B**) measurements of EZH2 and JMJD3 mRNA levels in spermatogonia after overexpression. (**C**) The protein levels of EZH2, JMJD3 and H3K27me3 in spermatogonia were detected and statistically analyzed by Western blot. The membrane is lysed prior to hybridization with the antibody and the image has been cropped for a more aesthetically pleasing display. The full- length blots can be obtained from Additional file 2: Fig S1

**Fig. 2:**
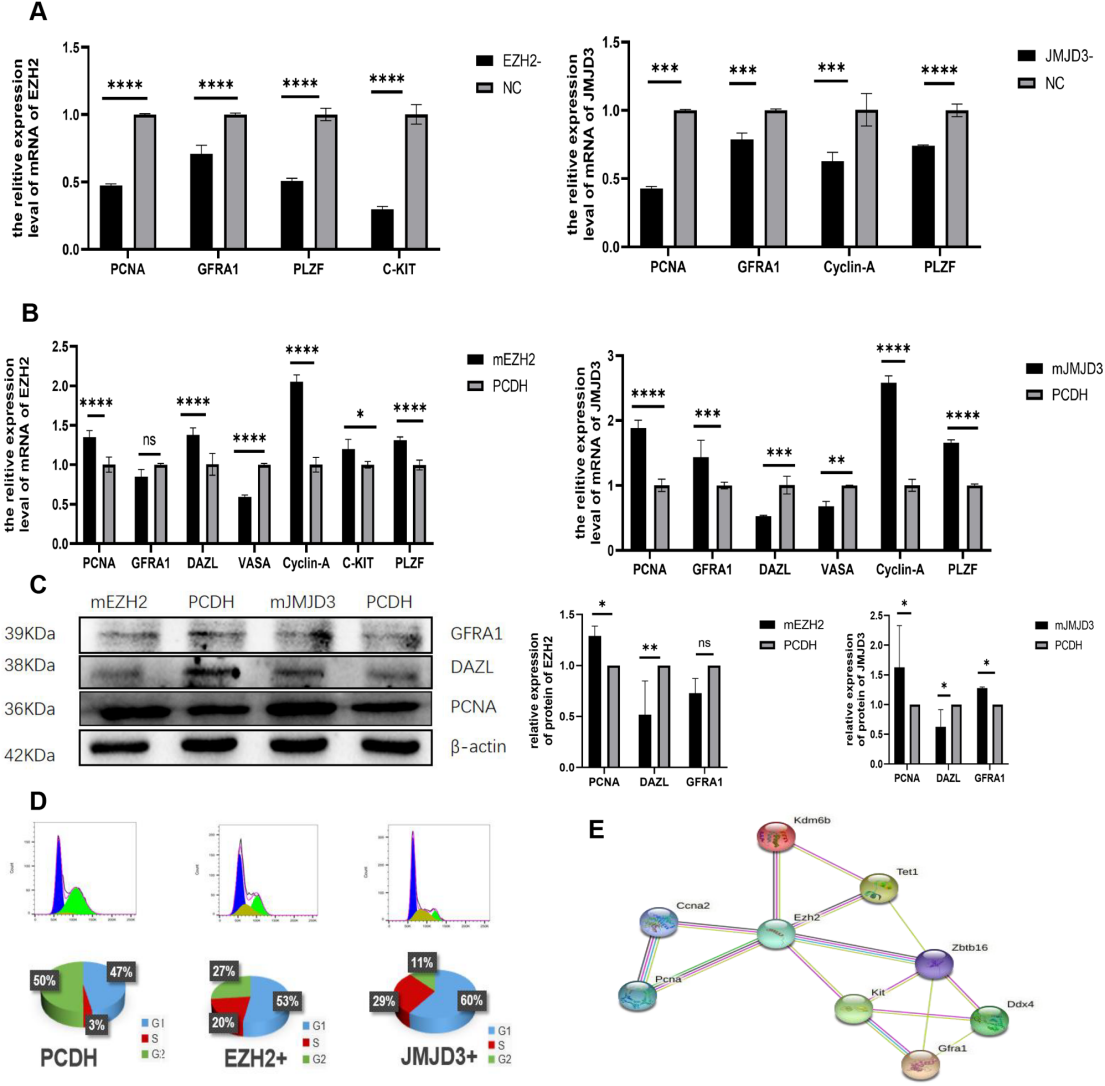
Effects of EZH2 interference or overexpression and JMJD3 interference or overexpression on self-renewal, proliferation and differentiation of spermatogonia. (**A**) The mRNA levels of PCNA, Cyclin-A, GFRA1, PLZF and C-KIT related to spermatogonia self-renewal and proliferation were changed after EZH2 and JMJD3 knockdown. (**B**) The expression of PCNA, cyclin-A, GFRA1, PLZF, C-KIT, DAZL and VASA was detected by qRT-PCR after EZH2 and JMJD3 overexpression. (**C**) The protein expression changes as well as statistical analysis of PCNA, DAZL and GFRA1 after EZH2 and JMJD3 overexpression. The membrane is lysed prior to hybridization with the antibody and the image has been cropped for a more aesthetically pleasing display. The full- length blots can be obtained from Additional file 2: Fig S2. (**D**) The cell cycle of EZH2 and JMJD3 overexpression cells was detected by flow cytometry. (**E**) Protein interaction network of EZH2, JMJD3 and spermatogonia self-renewal, proliferation and differentiation-related genes

To investigate the effects of JMJD3 on self-renewal, proliferation and differentiation in spermatogonium, qRT-PCR, Western blot and flow cytometry were used to detect both the specific siRNA of EZH2 and JMJD3 in spermatogonia, respectively, and the effects of overexpression modification on the biological characteristics of spermatogonia (Table 1). Firstly, according to the interference results of JMJD3-specific siRNA, the siRNA with the best inhibition effect was selected (Fig. 1A) and the expression of JMJD3 mRNA and protein levels were significantly increased in JMJD3-overexpressed cells (Fig. 1B and C). Besides, the findings of the biological properties assessment of JMJD3 on spermatogonia demonstrated that JMJD3 knockdown significantly reduced the mRNA levels of the PLZF and GFRA1 genes involved in spermatogonia self-renewal (Fig. 2A). On the contrary, the mRNA expression of self-renewal related genes PLZF and GFRA1 in JMJD3 overexpressed spermatogonia (Fig. 2B), as well as the protein expression of GFRA1, was increased (Fig. 2C). Flow cytometry showed that the proportion of S phase increased after JMJD3 overexpression (Fig. 2D). Simultaneously, the protein network interaction map revealed that JMJD3 was closely related to TET1 and GFRA1 self-renewal genes (Fig. 2E). JMJD3 functioned as a histone demethylase to play a role: for one thing, the overexpression of JMJD3 led to a significant decrease in the content of H3K27me3 (Fig. 1C) and for another, it regulated the expression of self-renewing genes such as GFRA1 and proliferation-related genes such as Cyclin-A; this indicated that JMJD3, as a major regulatory factor, was extraordinarily involved in the maintenance of self-renewal and proliferation of spermatogonia. Similarly, EZH2-specific siRNA resulted in significantly reduced mRNA expression levels (Fig. 1A), while EZH2 overexpression resulted in significantly increased mRNA and protein levels (Fig. 1B and C). Another control group: EZH2-specific siRNA inhibited the mRNA level expression of the self-proliferation-related gene PCNA (Fig. 2A), while mRNA and protein levels of PCNA related to promoting proliferation were all increased after EZH2 overexpression (Fig. 2B and C). Flow cytometry found that the proportion of the S phase increased after EZH2 overexpression (Fig. 2D). The interaction between EZH2 and the proliferation-related gene PCNA (Fig. 2E) was shown in the protein network interaction map. Nevertheless, the expression of the differentiation-promoting gene DAZL was upregulated at the mRNA level, whereas it was downregulated at the protein level (Fig. 2B and C). Meanwhile, the expression of differentiation-related gene KIT was down-regulated in EZH2-specific siRNA (Fig. 2A), but increased mRNA expression in EZH2 overexpressed cells (Fig. 2B). Additionally, the protein network interaction map showed that EZH2 interacts with KIT (Fig. 2E) as well. Considering the above-mentioned factors, EZH2 plays a major part in spermatogonium differentiation and JMJD3 has a significant regulatory role in promoting the self-renewal and proliferation of spermatogonium.

### TET1 overexpression forms a complex with JMJD3 to regulate the change of H3K27me3 content in spermatogonium

**Fig. 3:**
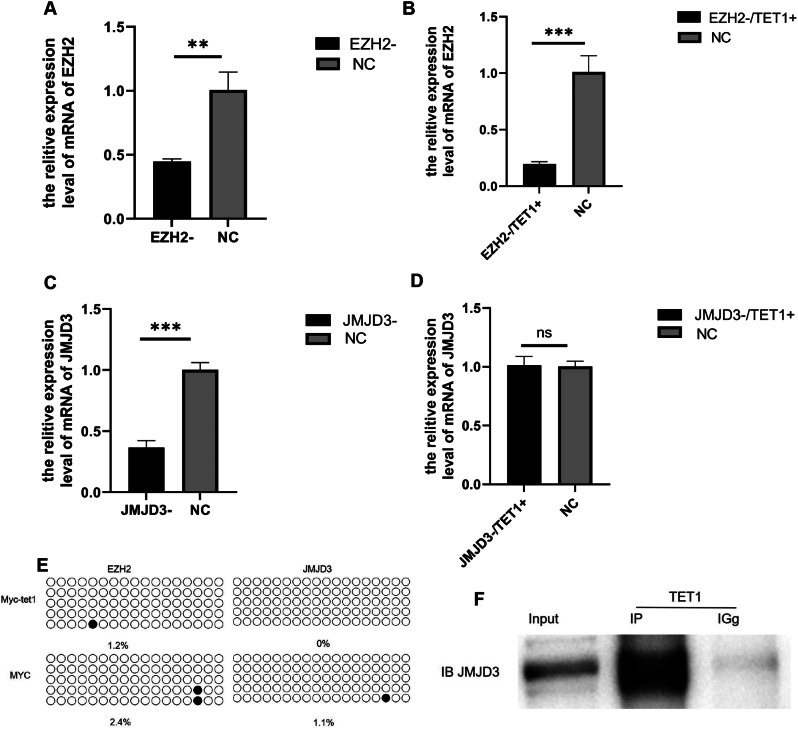
The manner in which EZH2, JMJD3 interacts with TET1 in spermatogonia. (**A**) The expression of EZH2 after EZH2 SiRNA in spermatogonia was detected by qRT-PCR. (**B**) qRT-PCR was used to detect the expression of EZH2 after co-transfection of EZH2 SiRNA and TET1 overexpression vector in spermatogonia. (**C**) The expression of JMJD3 in spermatogonia after JMJD3 SiRNA was detected by qRT-PCR. (**D**) qRT-PCR was used to detect the expression of JMJD3 after the co-transfection of JMJD3 SiRNA and TET1 overexpression vector in spermatogonia. (**E**) Bead plot of methylation sequencing results and methylation ratio after PCR amplification with primers designed for EZH2 and JMJD3(CpG-enriched region within the first 2000 bp of the promoter region), Filled circles represent methylated, empty circles represent unmethylated. (**F**) The JMJD3-TET1 protein interaction was detected by Co-IP technology. The membrane is lysed prior to hybridization with the antibody and the image has been cropped for a more aesthetically pleasing display. The full- length blots can be obtained from Additional file 2: Fig S3

Previous study showed that TET1 can promote the self-renewal and proliferation of spermatogonium [23, 28]. In order to investigate the regulatory relationship between TET1 and JMJD3 in the process of self-renewal and proliferation of spermatogonium, siRNA interference of EZH2 and JMJD3 was performed in TET1-overexpressing spermatogonia and non-TET modified spermatogonia in this study, which showed that the expression level of spermatogonium was significantly down-regulated under the SiRNA interference treatment of EZH2 (Fig. 3A). What’s more, under the co-modification of TET1 overexpression, the expression level of EZH2 in the EZH2 interference group was still decreased and TET1 had no effect on its expression (Fig. 3B). The expression level of JMJD3 in the JMJD3 interference group was obviously decreased (Fig. 3C) and there was no difference between the expression level and that of the TET1 overexpression co-treated group (Fig. 3D), indicating that the overexpression of TET1 can alleviate the influence of siRNA interference of JMJD3 on mRNA levels. Due to the overexpression of TET1, the transcription of JMJD3 mRNA was promoted to maintain a relatively high expression level. TET1-overexpressing cell samples were subjected to bisulfite detection of EZH2 and JMJD3 promoter regions, corresponding to CpG-enriched regions within the first 2000 bp of the promoter region (Table 2): The methylation region of EZH2 promoter region after TET1 modification was 1.2%, while the control group was 2.4%. The methylation region of JMJD3 after TET1 modification was 0%, while the control group was 1.1%. In contrast with the negative control group, the promoter regions of EZH2 and JMJD3 showed low methylation levels. Moreover, there was no significant difference before and after TET1 modification, indicating that TET1 did not modify EZH2 and JMJD3 through the DNA hydroxymethylation pathway (Fig. 3E). The co-IP detection of cell samples with both TET1 and JMJD3 overexpression showed direct protein interactions (Fig. 3F) between TET1 and JMJD3. From what has been discussed above, we can conclude that TET1 did not modify EZH2 and JMJD3 through the DNA hydroxymethylation pathway, but promoted the expression of JMJD3 and formed protein complex with JMJD3 to regulate the content of H3K27me3 in spermatogonium. Thus, the expression changes of H3K27me3-specific locus genes were regulated to regulate the self-renewal and proliferation of spermatogonium.

### TET1 coordinates with and H3K27me3 to target Pramel3 and promote its activation and expression

**Fig. 4:**
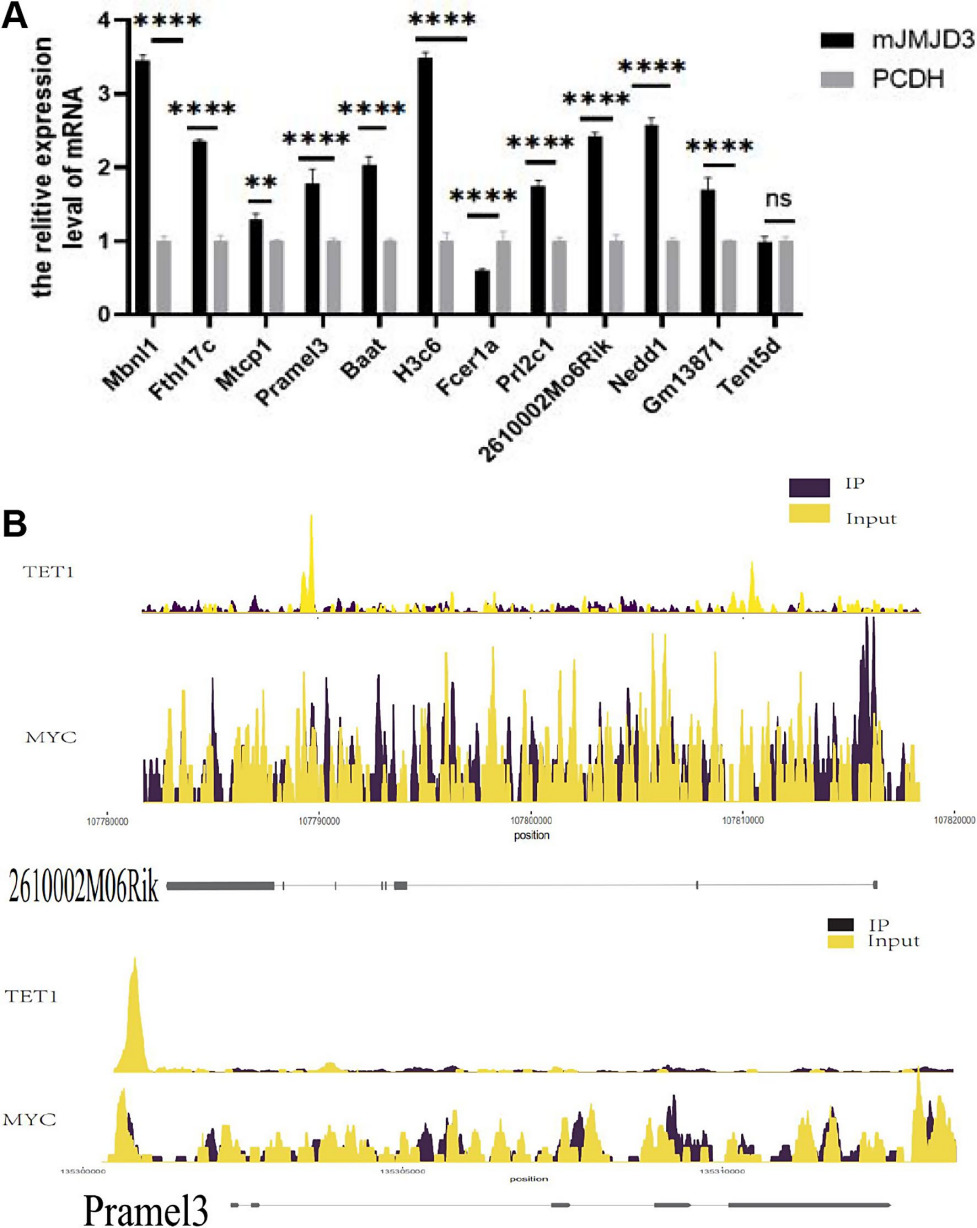
TET1 coordinates with H3K27me3 to target Pramel3 to promote its activation and expression. (**A**) qRT-PCR was used to detect the expression of genes enriched by Chip-seq after JMJD3 overexpression. (**B**) Peak plots of 2610002M06Rik and Pramel3 target genes obtained from TET1 overexpressing cells deposited with H3K27me3 antibody

**Fig. 5:**
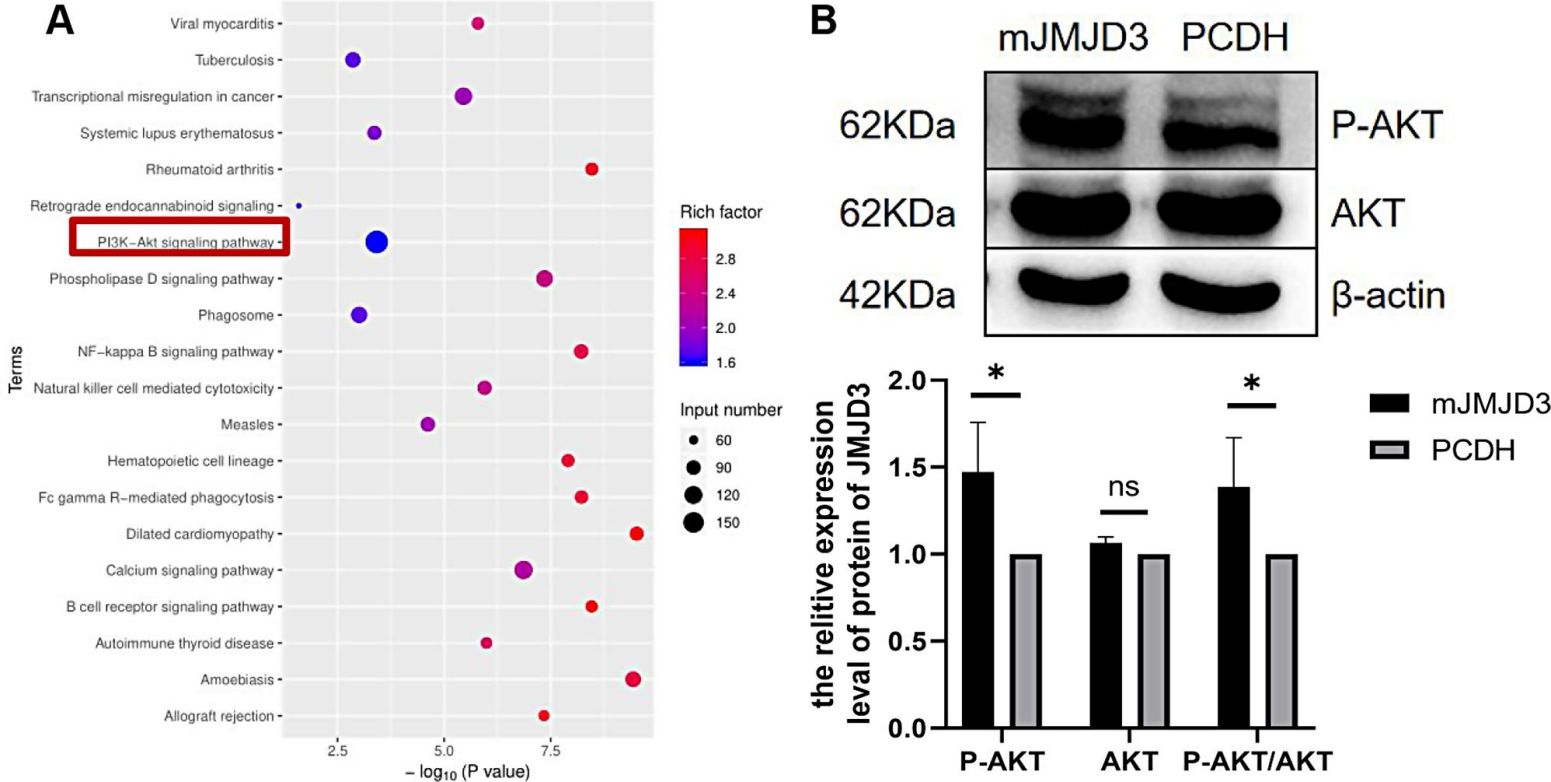
TET1-H3K27me3 regulated through PI3K-AKT pathway. (**A**) KEGG enrichment analysis. (**B**) Western blot was used to detect the protein expressions of AKT and P-AKT in the PI3K-AKT pathway after JMJD3 overexpression. The membrane is lysed prior to hybridization with the antibody and the image has been cropped for a more aesthetically pleasing display. The full- length blots can be obtained from Additional file 2: Fig. S4

To further identify the specific target genes that co-regulate H3K27me3 with JMJD3 after TET1 overexpression, we used Chip-seq sequencing and qRT-PCR techniques for detection. TET1 overexpressed cells and a control group were used for immunoprecipitation. Then, Chip-seq was performed with H3K27me3-specific antibodies to screen 12 target genes (Mbnl1, Fthl17c, Mtcp1, Pramel3, Baat, H3c6, Fcer1a, Prl2c1, 2610002M06Rik (Chmp1b2), Nedd1, Gm13871, Tent5d), among Mbnl1, Mtcp1, Pramel3, Baat, H3c6, Fcer1a, Prl2c1, 2610002M06Rik (Chmp1b2), Nedd1, Gm13871, Tent5d, whichMbnl1, Mtcp1, Pramel3, Nedd1, Tent5d may be related to spermatogonia [36–39]; the mRNA level was also verified at the same time. The expression levels of Mbnl1, Fthl17c, Mtcp1, Pramel3, Baat, H3c6, Prl2c1, 2610002M06Rik(Chmp1b2), Nedd1 and Gm13871 in JMJD3-overexpressed spermatogonium were all higher than those in the control group. Conversely, the expression of Fcer1a was lower than in the control group. In addition, there was no difference in the expression of Tent5d (Fig. 4A). 2610002M06Rik was involved in the regulation of the cell process and Pramel3 was expressed in testicular germ cells and regulated the development of spermatogonia. Therefore, taking target genes 2610002M06Rik and Pramel3 for example, we found that the peak difference map of their genes showed obvious differences. Compared with the control group, the content of H3K27me3 in the same locus of genes in the cell samples with overexpression of TET1 was significantly reduced, which indicated that overexpression of TET1 resulted in the decreased deposition of H3K27me3 in the Pramel3 gene, promoting its transcriptional activation. A significant up-regulation of Pramel3 was observed in JMJD3-overexpressing spermatogonia (Fig. 4B), indicating a coordinated role of Pramel3-TET1-JMJD3-H3K27me3 in spermatogonia self-renewal. KEGG results of Chip-seq showed that differential genes were significantly enriched in the PI3K-AKT pathway (Fig. 5A). In the PI3K-AKT pathway, the Ywhaq gene is involved in the regulation of the cell cycle and CDK6 is important for the progression of the G1 phase and G1/S transition of the cell cycle. PDGFC plays a vitally important role in regulating embryonic development and cell proliferation. Subsequently, Western blotting showed that the expression of P-AKT/AKT increased after JMJD3 overexpression (Fig. 5B). Overall, the results above demonstrate that Pramel3-TET1-JMJD3-H3K27me3 regulates spermatogonia self-renewal through the PI3K-AKT signaling pathway.

### Functional verification of JMJD3 in mice

**Fig. 6:**
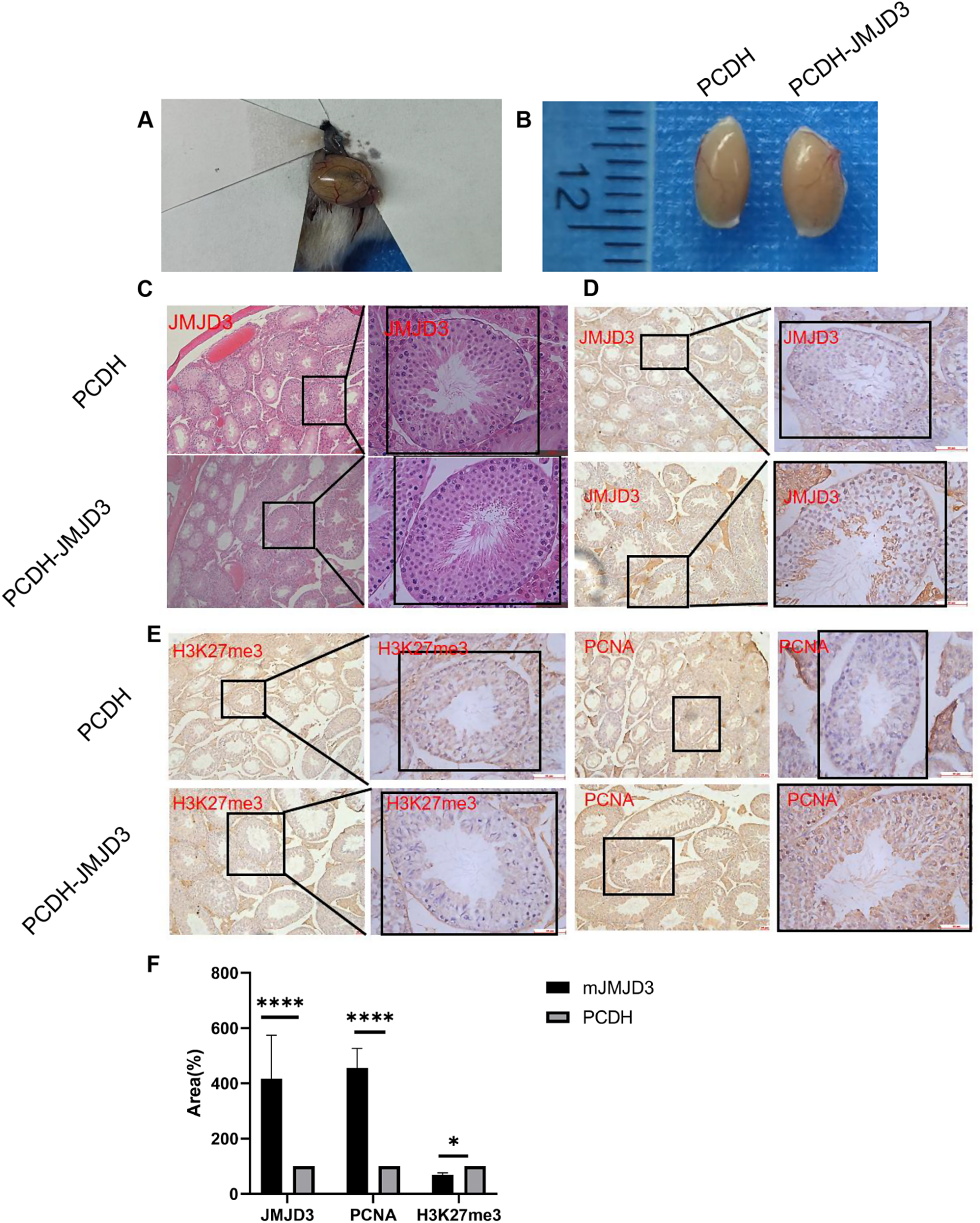
In vivo functional validation of JMJD3. (**A**) Spermatogenesis disorder model mice transplantation of control PCDH and JMJD3 Positive Cells. (**B**) Chart of comparison of control PCDH with testes transplanted with JMJD3 positive cells. (**C**) HE staining plot of JMJD3 overexpression versus control PCDH. (**D**) Expression of JMJD3 in testicular spermatogonia after overexpression of JMJD3. (**E**) Expression of H3K27me3 and PCNA in testicular spermatogonia after JMJD3 overexpression. (**F**) Statistical analysis of JMJD3 + cells/H3K27m3 + cells and PCNA + cells in immunohistochemistry. Scale bar = 50 μm. n = 3

The classic method to detect the biological characteristics of mouse spermatogonium is to transplant cells from mouse testicular models with spermatogenic defects (Fig. 6A). We found that the volume of the testis on the side with JMJD3-positive cells was significantly larger than that of the testis with negative control cells (Fig. 6B) after the mouse testis were surgically removed 8 weeks later. HE staining of the tissue sections (Fig. 6C) showed that only part of the spermatogenic tubules were successfully transplanted due to scattered and multi-point injection of cells, so there are cells distributed within the seminiferous tubules. In addition, some seminiferous tubules were still vacuolated in both groups because no cells were injected into some seminiferous tubules. Observing the distribution of spermatogenic epithelial cells in the cross-section of the curved seminiferous duct, the results showed that there were more cells on the side of the JMJD3-positive cells than on the side of the negative control cells and the cell density was larger as well, but the cells were not evenly distributed, growing freely in the lumen of the curved seminiferous duct. Furthermore, compared with the control cells, more cells were in the primary spermatocyte stage and the new spermatogenesis process started faster as well, which proved that JMJD3-positive cells were especially capable of proliferating. Immunohistochemical analysis (Fig. 6D, E and F) of JMJD3, H3K27me3 and proliferation-related gene PCNA were performed on testicular tissues of the two groups. Naturally, it has been proved that histone epigenetic modification was involved in the long-term growth process of cells in a mouse testis model from the protein level and that JMJD3 overexpression can promote both self-renewal and proliferation of mouse spermatogonia has been certified in depth.

## Discussion

The latest research reveals that approximately 15% of couples in their reproductive years worldwide encounter infertility issues, with nearly half of these cases attributed to male infertility. Among male infertility patients, a significant proportion is caused by spermatogenic disorders [40]. Therefore, the identification and characterization of genes associated with spermatogenic disorders are crucial for comprehending the genetic etiology of male infertility and elucidating its underlying mechanisms. In this study, we have discovered that the co-regulation of TET1 and H3K27me3 plays a crucial role in governing the self-renewal, proliferation and differentiation of spermatogonia. In order to validate this finding, we initially overexpressed the methylase EZH2 and demethylase JMJD3 of H3K27me3 in spermatogonia, subsequently assessing alterations in their mRNA and protein levels to examine changes in their biological characteristics. In the meantime, the protein interaction between TET1 and JMJD3 was concurrently validated. Likewise, Chip-seq sequencing of H3K27me3 was conducted on cell samples overexpressing TET1 to determine its mechanistic underpinnings. As shown by the experimental results, the content of H3K27me3 in TET1 overexpressed spermatogonia decreased because TET1 promoted the function of JMJD3 by forming a complex with JMJD3; also, the precipitation of H3K27me3 in target genes such as Pramel3 up-regulated the expression of Pramel3 to achieve epigenetic regulation of spermatogonia.

This study found that JMJD3 plays a leading role in the self-renewal of spermatogonia: The overexpression of JMJD3 as a demethylase can decrease the level of H3K27me3 and facilitate gene transcriptional regulation [9]. After the overexpression of JMJD3, the detection of genes related to the self-renewal of spermatogonia found that the expression of GFRA1 gene related to self-renewal was up-regulated, indicating that JMJD3 promoted the self-renewal of spermatogonia cells by regulating the methylation state of H3K27me3 to maintain the dynamic balance of spermatogonia cells. Some studies have discovered that EZH2 exerts a vital role in the proliferation of spermatogonocytes [26]. Also, epigenetic modifications encompass DNA and histone modifications, with intricate crosstalk between them facilitating mutual regulation and collaborative functioning [41]. As a DNA hydroxymethylase, TET1 usually regulates transcription by influencing the methylation status of the transcription factor promoter region. The promoter methylation levels of JMJD3 and EZH2 in TET1-modified spermatogonia were also very low, with no significant difference compared to the control group, indicating that TET1 acts on JMJD3 as an interacting protein. The interaction between TET1 and JMJD3 proteins was confirmed by Co-IP experiments, suggesting the formation of a complex to exert its effects on H3K27me3 [18]. When TET1 interacts with JMJD3, it can enhance demethylation to inhibit the increase in H3K27me3 content, so that the reduction of H3K27me3 deposition at the gene locus is undoubtedly evident and makes genes at relevant loci in an activated state to further regulate the self-renewal of spermatogonia. The formation of a TET1/PRC2 complex targeting H3K27me3 has been previously demonstrated [21]. More specifically, it has been reported that the gene promoter TET1 overlaps with PRC2, thereby influencing H3K27me3 [42].

The Chip-seq analysis of H3K27me3 was conducted on cells overexpressing TET1, resulting in the identification of twelve potential target genes: Mbnl1, Fthl17c, Mtcp1, Pramel3, Baat, H3c6, Fcer1a, Prl2c1, 2610002M06Rik (Chmp1b2), Nedd1, Gm13871 and Tent5d. Subsequently, three specific target genes (2610002M06Rik, Pramel3 and Prl2c1) were selected and Pramel3 was expressed in all stages of spermatogenesis [29]. Therefore, it is speculated that the interaction between TET1 and H3K27me3 promotes the up-regulation of Pramel3 expression to regulate the dynamic balance of spermatogonia self-renewal. Moreover, differential genes are enriched in the PI3K-AKT pathway, which is related to cell fertility and promotes the self-renewal, proliferation and differentiation of spermatogonia through cell signal transduction. The literature reports that androgens enhance HIF1α protein levels and induce JMJD3 expression in mouse spermatogonia via the PI3K-Akt signaling pathway [7]. Some data illustrate that the Akt pathway is implicated in JMJD3 expression in PDGF-stimulated FLS [43]. It has been reported that PRAME interacts with retinoic acid receptors (RARs) to inhibit the RA signaling pathway for the transcriptional regulation of cancer cells [30]. The significance of RAR-mediated signaling in the initiation of spermatogenesis has been demonstrated in both germ cells and Sertoli cells [31, 32]. Studies have shown that PRAME induces the proliferation, differentiation, migration and invasion of LSCC cells depending on activation of the PI3K/AKT/mTOR signaling pathway [44]. The CDK6 gene was screened within the PI3K-AKT pathway and its up-regulation facilitated the transition from the G1 phase to the S phase, thereby promoting cellular proliferation. Previous studies have demonstrated the crucial involvement of CDK6 in cancer cell proliferation [45]. PI3K inhibitors have been found to inhibit the proliferation of endometrial cancer cells by decreasing CDK6, increasing p27 and subsequently inducing G1 phase arrest [46].

## Conclusion

In conclusion, the regulatory mechanism of TET1-H3K27me3 on self-renewal in spermatogonia was investigated using molecular biology and high-throughput sequencing techniques, confirming that TET1 and JMJD3 co-regulate the levels of H3K27me3 through the formation of a protein complex. Additionally, the target gene Pramel3 for TET1-H3K27me3 was identified. On top of that, the regulatory axis of TET1-JMJD3-H3K27me3-Pramel3 is utilized for the regulation of spermatogonia self-renewal and proliferation, providing a scientific foundation for investigating genes associated with spermatogenesis and disorders as well as their pathogenesis.

### Electronic supplementary material

Below is the link to the electronic supplementary material.

**Additional file 1:** Lentiviral transduction and positive cell clone screen. (**A**) Lentiviral vector plasmid mapping of PCDHEZH2 and PCDH-JMJD3. (**B**) Screening for monoclonal positive cells. Scale bar = 50 μm. n = 3

**Additional file 2: Fig. S1.** Image of three replicate trials of original protein blotting of Fig. 1C. (**A, B, C**) Full-length blot of EZH2 and β-actin protein expression level. The red marker indicates the position of the crop. (**D, E, F**) Full-length blot of JMJD3 and β-actin protein expression level. The red marker indicates the position of the crop (**G, H, I**) Full-length blot of H3K27me3 and H3 protein expression level. The red marker indicates the position of the crop. **Fig. S2.** Image of three replicate trials of original protein blotting of Fig. 2C. (**A, B, C**) Full-length blot of GFRA1 and β-actin protein expression level. The red marker indicates the position of the crop. (**D, E, F**) Full-length blot of DAZL and β-actin protein expression level. The red marker indicates the position of the crop (**G, H, I**) Full-length blot of 13 PCNA and β-actin protein expression level. The red marker indicates the position of the crop. **Fig. S3.** Image of original protein blotting of Fig. 3F. (**A, B, C**) Full-length blot of three replicate trials of IB JMJD3 protein expression level. The red marker indicates the position of the crop. (**D**) Full-length blot of IB TET1 protein expression level. The red marker indicates the position of the crop. **Fig. S4.** Image of three replicate trials of original protein blotting of Fig. 5B. (**A, B, C**) Full-length blot of P-AKT, AKT and β-actin protein expression level. The red marker indicates the position of the crop

## Data Availability

The datasets presented in this study can be found in online repositories. The names of the repository/repositories and accession number(s) can be found below: GSE233150.
