# Supplementary material for: JMJD3 regulate H3K27me3 modification via interacting directly with TET1 to affect spermatogonia self-renewal and proliferation
Source: BMC Genomics. 2024 Feb 29;25:225. doi: 10.1186/s12864-024-10120-9 (PMC10905883; doi:10.1186/s12864-024-10120-9)
Supplement: Supplementary file 1 — Additional file 1: Lentiviral transduction and positive cell clone screen. (A) Lentiviral vector plasmid mapping of PCDHEZH2 and PCDH-JMJD3. (B) Screening for monoclonal positive cells. Scale bar = 50 μm. n = 3 [file 12864_2024_10120_MOESM1_ESM.pdf]

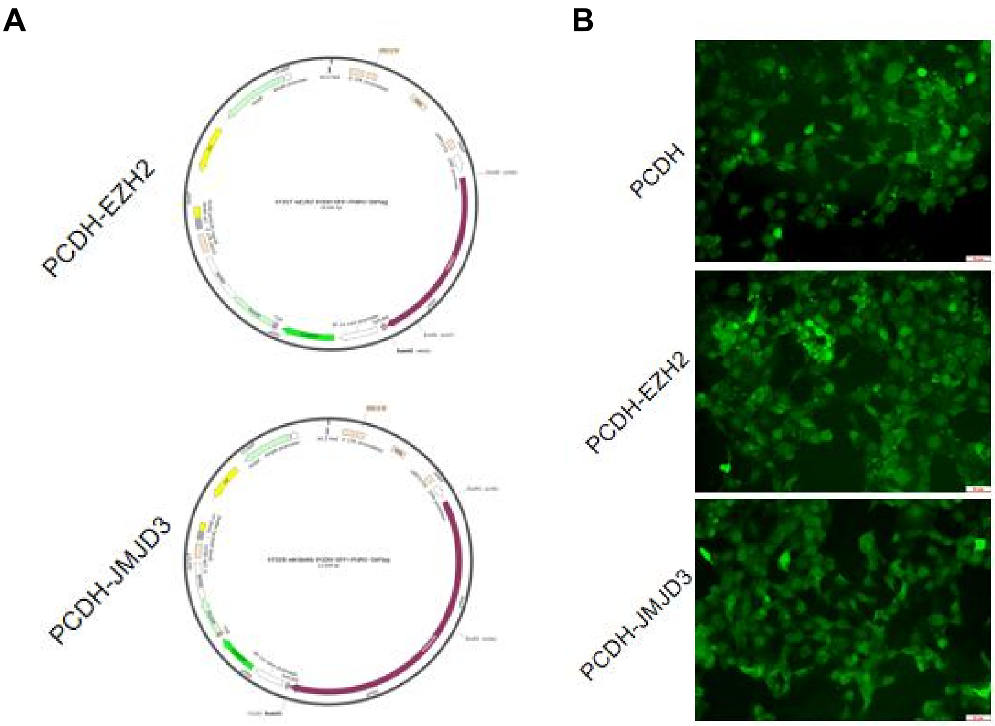

Additional file 1
Lentiviral transduction and positive cell clone screen. (A) Lentiviral vector plasmid mapping of PCDH-EZH2 and PCDH-JMJD3. (B) Screening for monoclonal positive cells. Scale bar=50μm. n=3.
