# Supplementary material for: JMJD3 regulate H3K27me3 modification via interacting directly with TET1 to affect spermatogonia self-renewal and proliferation
Source: BMC Genomics. 2024 Feb 29;25:225. doi: 10.1186/s12864-024-10120-9 (PMC10905883; doi:10.1186/s12864-024-10120-9)
Supplement: Supplementary file 2 — Additional file 2: Fig. S1. Image of three replicate trials of original protein blotting of Fig. 1C. (A, B, C) Full-length blot of EZH2 and β-actin protein expression level. The red marker indicates the position of the crop. (D, E, F) Full-length blot of JMJD3 and β-actin protein expression level. The red marker indicates the position of the crop (G, H, I) Full-length blot of H3K27me3 and H3 protein expression level. The red marker indicates the position of the crop. Fig. S2. Image of three replicate trials of original protein blotting of Fig. 2C. (A, B, C) Full-length blot of GFRA1 and β-actin protein expression level. The red marker indicates the position of the crop. (D, E, F) Full-length blot of DAZL and β-actin protein expression level. The red marker indicates the position of the crop (G, H, I) Full-length blot of 13 PCNA and β-actin protein expression level. The red marker indicates the position of the crop. Fig. S3. Image of original protein blotting of Fig. 3F. (A, B, C) Full-length blot of three replicate trials of IB JMJD3 protein expression level. The red marker indicates the position of the crop. (D) Full-length blot of IB TET1 protein expression level. The red marker indicates the position of the crop. Fig. S4. Image of three replicate trials of original protein blotting of Fig. 5B. (A, B, C) Full-length blot of P-AKT, AKT and β-actin protein expression level. The red marker indicates the position of the crop [file 12864_2024_10120_MOESM2_ESM.pdf]

Additional file 2

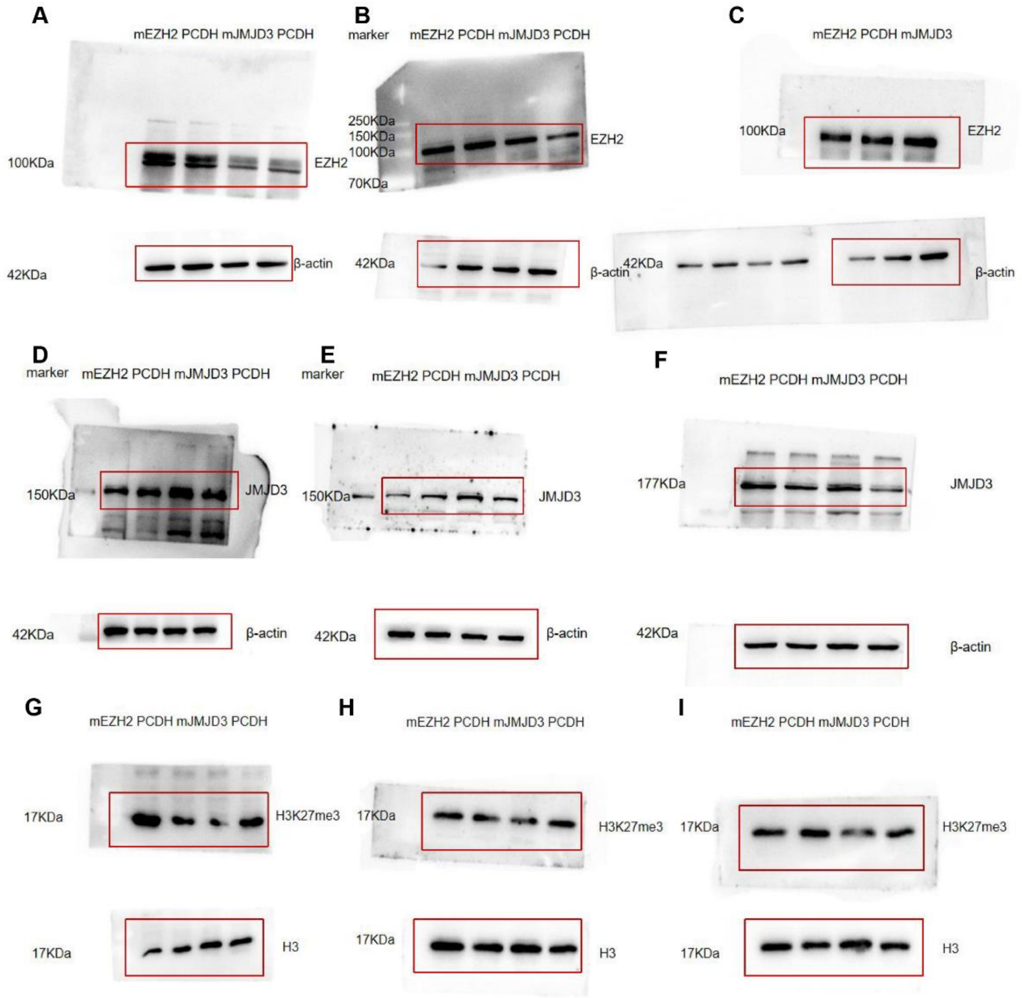

Fig S1 Image of three replicate trials of original protein blotting of Fig 1C.

(A, B, C) Full-length blot of EZH2 and β-actin protein expression level. The red marker indicates the position of the crop. (D, E, F) Full-length blot of JMJD3 and β-actin protein expression level. The red marker indicates the position of the crop (G, H, I) Full-length blot of H3K27me3 and H3 protein expression level. The red marker indicates the position of the crop.

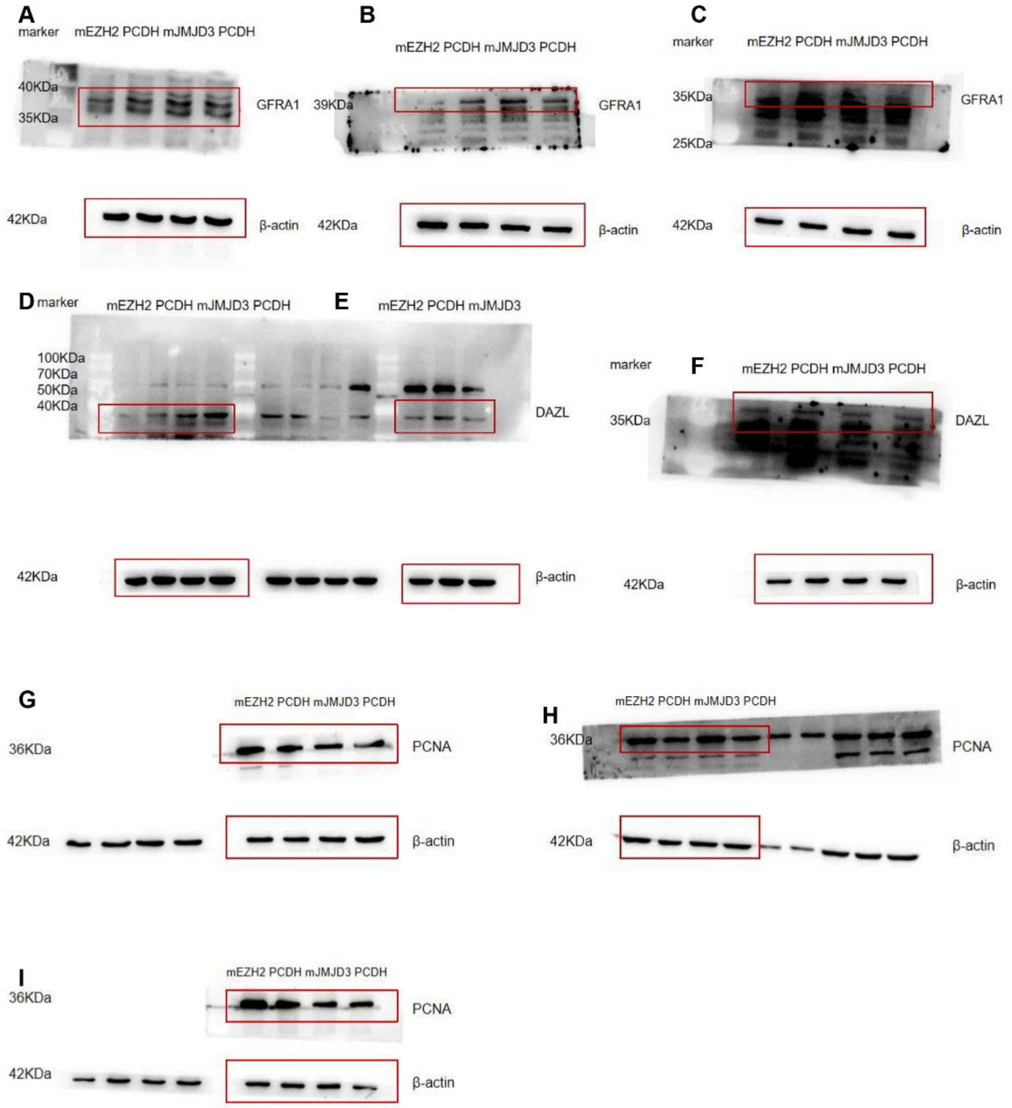

Fig S2 Image of three replicate trials of original protein blotting of Fig 2C.
(A, B, C) Full-length blot of GFRA1 and β-actin protein expression level. The red marker indicates the position of the crop. (D, E, F) Full-length blot of DAZL and β-actin protein expression level. The red marker indicates the position of the crop (G, H, I) Full-length blot of PCNA and β-actin protein expression level. The red marker indicates the position of the crop.

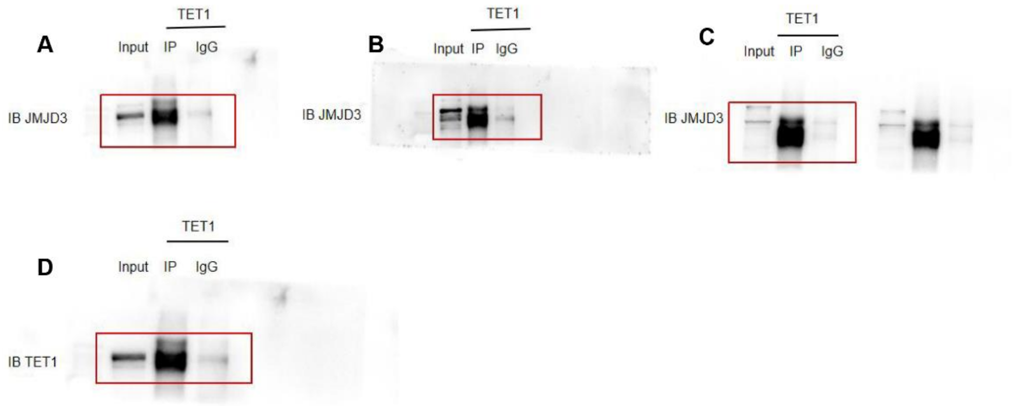

Fig S3 Image of original protein blotting of Fig3F.

(A, B, C) Full-length blot of three replicate trials of IB JMJD3 protein expression level. The red marker indicates the position of the crop. (D) Full-length blot of IB TET1 protein expression level. The red marker indicates the position of the crop.

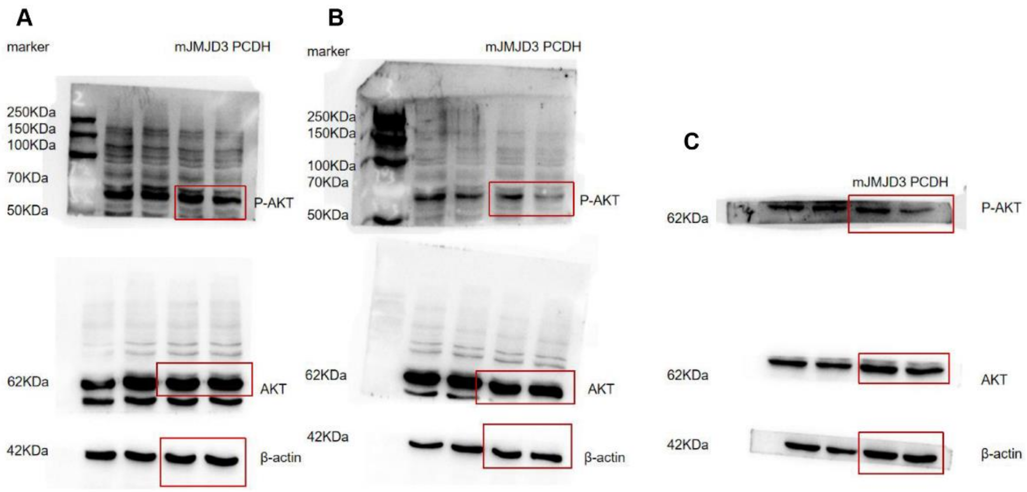

Fig S4 Image of three replicate trials of original protein blotting of Fig 5B.

(A, B, C) Full-length blot of P-AKT, AKT and β-actin protein expression level. The red marker indicates the position of the crop.
